# Discrimination and Social Isolation Among African Americans Across the Lifespan

**DOI:** 10.1093/geroni/igaa057.1934

**Published:** 2020-12-16

**Authors:** Ann Nguyen, Harry Taylor

**Affiliations:** 1 Case Western Reserve University, Cleveland, Ohio, United States; 2 Duke University Medical Center, Durham, North Carolina, United States

## Abstract

Social isolation is associated with a wide range of health problems, including early mortality. However, little is known about the risk factors for social isolation specifically among African Americans. This study examined 1) the associations between discrimination and objective and subjective social isolation and 2) how these associations vary by age in a nationally representative sample of African American adults from the National Survey of American Life (N=3570). Multinomial logistic regression analyses indicated that discrimination was positively associated with being subjectively isolated from friends only and family only. Discrimination did not predict objective isolation. A significant interaction revealed that the association between discrimination and subjective isolation from friends only varied by age, with older adults being most vulnerable to the effects of discrimination. These findings argue for a more nuanced and systematic investigation of the detrimental effects of discrimination on older African Americans’ social relationships, especially perceptions of relationships.

